# The Effect of Forced Language Switching during Divergent Thinking: A Study on Bilinguals’ Originality of Ideas

**DOI:** 10.3389/fpsyg.2017.02086

**Published:** 2017-12-05

**Authors:** Martin Storme, Pinar Çelik, Ana Camargo, Boris Forthmann, Heinz Holling, Todd Lubart

**Affiliations:** ^1^Laboratoire Adaptations Travail Individu, Université Paris Descartes, Paris, France; ^2^Economics and Management, Centre Emile Bernheim, Université Libre de Bruxelles, Brussels, Belgium; ^3^Department of Psychology, University of Münster, Münster, Germany

**Keywords:** bilingualism, language switching, divergent thinking, originality of ideas

## Abstract

In the present study we experimentally manipulated language switching among bilinguals who indicated to be more or less habitual language switchers in daily life. Our aim was to investigate the impact of forced language switching on originality of produced ideas during divergent thinking, conditional on the level of habitual language switching. A sample of bilinguals was randomly assigned to perform alternate uses tasks (AUT’s), which explicitly required them to either switch languages, or to use only one language while performing the tasks. We found that those who were instructed to switch languages during the AUT’s were able to generate ideas that were on average more original, than those who were instructed to use only one language during the AUT’s, but only at higher levels of habitual language switching. At low levels of habitual language switching, the effect reversed, and participants who were instructed to use only one language found ideas that were on average more original, than participants who were required to switch languages during the AUT’s. Implications and limitations are discussed.

## Introduction

Language switching or code switching – i.e., alternating between and mixing multiple languages in conversation – is common practice among many bilinguals (Lin, 2013). Literature increasingly suggests that speaking more than one language enhances general cognitive abilities, such as creative cognition (Ricciardelli, 1992; Lee and Kim, 2011). A recent report of the European Commission identified more than 200 articles demonstrating a connection between bilingualism and creative potential (European Commission, 2009; Hommel et al., 2011), with recent studies suggesting a link between language switching and creative cognition (e.g., Wei and Wu, 2009). These studies suggest a positive relation between stable individual differences in daily language switching and creativity. Based on these findings the question emerges whether bilinguals should be specifically encouraged to switch languages as much as possible to be more creative. It is not known to what extent forced language switching would be beneficial to creativity, given large variations between bilinguals regarding their daily habits of language switching. Therefore, in the current study we experimentally manipulated language switching in an idea generation task among bilinguals’ with varying levels of daily language switching habits, and tested the originality of their ideas. As we will outline below, we reasoned that whether forced language switching increases creativity might crucially depend on bilinguals’ daily habits regarding language switching.

### Bilingualism, Language Switching, and Cognitive Functioning

The link between bilingualism and enhanced cognitive functions is currently a debated issue in the literature. Some studies found, for example, that being able to speak multiple languages is not particularly associated with enhanced cognitive executive control functions (e.g., Paap et al., 2017; Ross and Melinger, 2017). Other studies show that bilingualism is positively associated with cognitive executive functions (e.g., Colzato et al., 2008; Bialystok, 2011). For example, Bialystok and Viswanathan (2009) showed that inhibitory control and shifting – i.e., cognitive flexibility, which is considered the result of basic cognitive executive functions such as inhibition, updating and shifting (Miyake et al., 2000) – were better developed in bilinguals than in monolinguals. Carlson and Meltzoff (2008) found that early bilingual children – i.e., individuals who have two mother tongues – compared to late bilingual and monolingual children, outperformed the latter two groups especially in measures that required working memory and the inhibition of distracting information.

In the literature, the enhanced flexibility and cognitive executive functions of bilinguals – especially of the ‘early’ ones – is ascribed to bilinguals’ constant need to monitor and control the non-target language when conversing, which is thought to act as an almost constant exercise of their cognitive executive functions (Rodriguez-Fornells et al., 2006; Ye and Zhou, 2009; Bialystok, 2011). Indeed, for early bilinguals it seems that both their language systems are simultaneously active as a default (Starreveld et al., 2014). These individuals, even when speaking one language, may think in two languages, and thus be in a more or less continuous language switching state.

The debate on the purported advantages of bilinguals on executive functions is paralleled in practice as well, with many schools actively discouraging language switching, believing that it is detrimental to group cohesion, communication and language development (Ferguson, 2003; Wei and Wu, 2009; Lin, 2013). Language switching seems only encouraged by educators in specific contexts, for example when learning a second language (Moore, 2002; Lin, 2013).

However, some authors have suggested that the increased cognitive flexibility of bilinguals has an additional benefit for the cognitive functioning of bilinguals; it is believed to stimulate their creativity. Indeed, cognitive flexibility lies at the core of the ability to think outside of usual cognitive patterns and overcome functional fixedness (Guilford, 1967). In the literature, cognitive flexibility, and ‘out of the box thinking’ is conceptualized as being one of the core components of creative cognition (Beghetto and Kaufman, 2007), and several empirical studies have shown that flexibility is positively associated with creative achievement (Carson et al., 2005).

Importantly, many studies have shown a creative advantage of bilinguals over monolinguals, and of early bilinguals over late bilinguals (for a review, see Kharkhurin, 2012). Bilingualism may thus over time result in enhanced creativity – presumably fueled by the constant cognitive monitoring of distracting language systems which, over time, enhances cognitive flexibility and creativity (Rodriguez-Fornells et al., 2006; Ye and Zhou, 2009; Bialystok, 2011).

Taken together, previous literature suggests that accumulating language switching experiences might over time have a positive effect on bilinguals’ creativity. Does this also mean that bilinguals should be specifically encouraged to switch languages as much as possible to be more creative? This is an important question because it may help shape language use policies in bilingual or multilingual schools, but also in organizations in general that seek to improve their creative output.

We suggest that the answer to this question depends on the extent to which individuals are used to switching languages in daily life. Because for individuals who are used to switching languages regularly, switching languages could be considered their normal state, each single episode of language switching may stimulate their creativity, while each single language use episode may hinder their creativity. For example, Prior and Gollan (2011), showed that bilinguals who were used to switching languages in daily life exhibited less task switching costs than monolinguals. The authors speculated that daily language switching may be crucial to the advantages of bilingualism regarding general task switching abilities. Likewise, Soveri et al. (2011), also reported that among early bilinguals higher rates of everyday language switches were positively associated with performance in a set shifting task. Finally, a more recent study conducted by Kharkhurin and Wei (2015) showed, in verbal and graphical divergent thinking tasks, that bilinguals who switch languages more often found more ideas and also ideas that were more original, than their non-habitual counterparts.

For those who are less used to switching languages in their daily life, forced episodes of language switching are likely to come with a cognitive cost (Gollan and Ferreira, 2009), especially when switching from L2 to L1 (Meuter and Allport, 1999; Thomas and Allport, 2000). As a consequence, their creativity might be hindered when being forced to switch languages.

### Current Study

In the present study we investigate the effect of language switching on creative production in a divergent thinking Alternate Uses Task (AUT, Guilford, 1967). Divergent thinking can be defined as the process that allows people to generate as many responses as possible to a given problem (Guilford, 1967). In the AUT people are presented with an everyday object, such as a brick, and asked to generate as many uses for the brick as they can think of. The idea is that in divergent thinking relatively loosely controlled, spontaneous and associative memory searches alternate with more top–down cognitively controlled processes for the selection of unique and creative ideas. Divergent thinking tasks are usually scored in terms of fluency (number of ideas) and originality (uniqueness of ideas) (Mouchiroud and Lubart, 2001).

We designed a study in which we explicitly asked bilinguals, to either use only one language in the *non-switch* AUT condition, or to use both languages and alternate between them in the *switch* AUT condition, while generating ideas on how to use everyday objects. Participants were randomly assigned to these conditions. We then tested the effect of switching, compared to not switching during the AUT’s, on originality of the generated ideas. Importantly, we tested the moderating effect of the level of daily language switching on the relationship between switching languages in the task and the originality of ideas found during the task. We had several hypotheses. First, we expected an interaction between the type of AUT task (switch vs. non-switch AUT) and level of daily language switching. Specifically, we expected that at high levels of habitual language switching, there would be a positive effect of switching languages during the task on the originality of ideas. At low levels of habitual language switching, we hypothesized that there would be a negative effect of switching languages during the task on the originality of ideas.

In divergent thinking tasks it is common to assess the number or ideas, i.e., fluency, in addition to the originality of ideas. Often the two are positively correlated, and originality scores are typically analyzed controlling for fluency (Ritter et al., 2012). Therefore, we also measured fluency. Regarding this measure, we expected that compared to the non-switch condition, the switch condition would result in a lower the number of ideas generated in the task, i.e., lower fluency. This is because language switching has been shown to increase the time needed to perform tasks (Monsell, 2003), and multiple language activation reduces fluency (e.g., Bergmann et al., 2015).

## Materials and Methods

### Participants

The study involved 104 participants (55 females, *M*_age_ = 21.42 years, *SD* = 2.63) participated in the study. All participants indicated French as their mother tongue (i.e., first language, L1). Participants indicated English, Spanish, German, or Italian as their L2. To assess the extent to which participants engage in language switching in daily life we asked the question: “How often do you switch back and forth to L2 (or L1), when speaking L1 (or L2).” Participants answered this question on a six-point Likert scale ranging from *Never* (1), *Once a year* (2), *Several times a year* (3), *Once a month* (4), *Once a week* (5), *Every day* (6). In our sample, the average language switching frequency was *M* = 4.34 (*SD* = 1.85). The distribution was slightly platykurtic (kurtosis = 2.50) and left skewed (skewness = -0.71), meaning that there tended to be more participants switching often, than participants switching less often.

### Material

#### Divergent Thinking Tasks

Creativity was assessed with the AUT (Guilford, 1967). Participants completed three unusual uses tasks for the following objects: a spoon, a jump rope, and a plastic water bottle. Each task had a duration of 2 min. Participants were randomly assigned to one of the two conditions (*N*_switch_ = 52, *N*_non-switch_ = 52); those in the non-switch condition were asked to complete the AUT’s using only French (L1). Those in the switch condition were instructed to begin by writing an idea in French, followed by writing an idea in L2, and to keep alternating between languages during the 2 min that each task lasted. To aid the scoring of originality, those in the switch language condition were asked to translate their ideas written in L2 back to French after they were finished with all three tasks. We specifically instructed participants to generate as many *creative* and *unique* ideas as possible. When instructions of a divergent thinking task explicitly ask participants to come up with many creative and unique ideas (Forthmann et al., 2016), the task likely requires more cognitive flexibility, such as switches of search strategies and the inhibition of unoriginal ideas, than a task in which participants are simply asked to generate as many ideas as possible.

Originality scores were obtained via uniqueness scoring (Mouchiroud and Lubart, 2001) for each generated use in the AUT’s. Participants received a score of 5 for an idea when <5% of the other participants had come up with the same idea (i.e., very original), a 4 when approximately 10% had the same idea, a score of 3 when 20% of the participants had written the same response, a score of 2 when around 30% had written the same response, a score of 1 when >50% of participants had produced the same idea (i.e., not original at all). Furthermore, fluency scores (a count variable, summing all distinct ideas per object) were assigned to each participant. Originality and fluency scores were averaged across all items. The reliability of the originality score (α = 0.77) and the fluency score (α = 0.86) was satisfactory. In our sample the overall bivariate correlation between fluency and originality scores was 0.32, which is a common finding in the literature (Mouchiroud and Lubart, 2001).

### Procedure

Participants were informed that they would be participating in a study about the “effects of different university majors on creativity,” without revealing the specific objective of the study. They performed the three divergent thinking tasks always in the same order. Additional questions including demographic information, as well as the question about daily language switching were presented at the end. All instructions were given in French.

### Data Analysis

The analysis was conducted in two steps. We first analyzed the effect of the manipulation in interaction with the extent to which participants switch languages in daily life on fluency scores. Then, we analyzed the effect of the manipulation in interaction with the extent to which participants switch languages in daily life on originality scores. Because we used a between-subject design, we used multiple linear regressions to test our hypotheses. In all analyses, quantitative predictors were normalized, and the AUT condition was effect coded as follows: -0.5 = non-switch, 0.5 = switch.

## Results

**FIGURE 1:**
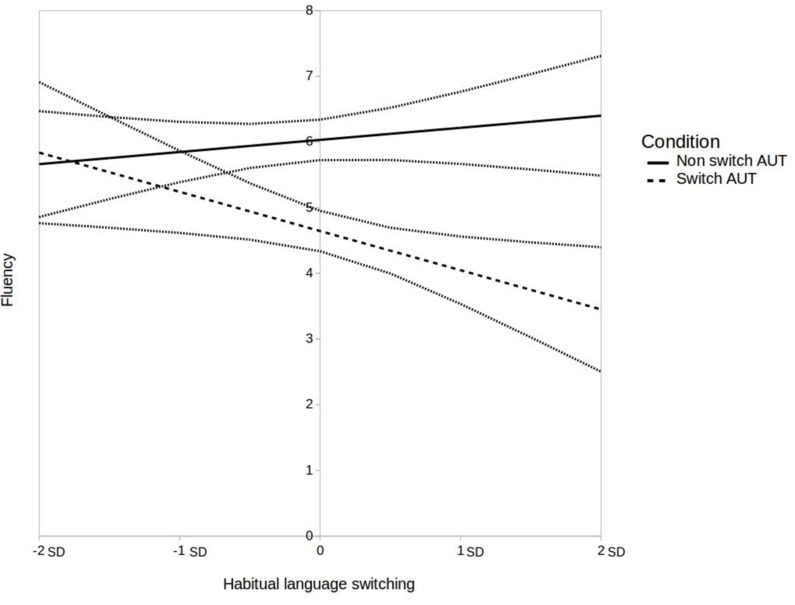
Interaction of language switching condition and habitual language switching on fluency scores. 95% confidence intervals are represented by gray lines.

A regression analysis with AUT condition and habitual language switching as predictors of fluency scores revealed a significant interaction effect, *B* = -0.78, *SE* = 0.33, *t* = 2.39, *p* < 0.05. The interaction effect is shown in **Figure 1**. We conducted exploratory simple slope analyses to further investigate the interaction effect, to which we applied a Bonferroni correction and set the significance threshold at 0.01. We found that in the switch condition, bilinguals who are used to switching languages (i.e., at +2 SD from the mean) tended to have lower fluency scores than their counterparts in the non-switch condition, *B* = -2.95, *SE* = 0.71, *t* = - 4.13, *p* < 0.001.

Because of the correlation between fluency and originality scores (*r* = 0.32), and because fluency appeared affected by the interaction between AUT condition and level of habitual language switching, we controlled for fluency in the analyses on originality^1^.

Estimates of the models with (Model B) and without fluency (Model A) as a control variable are presented in **Table 1**. In the following, we report analyses related to Model B.

**Table 1:** Estimates of the regression models predicting originality.

	Model A	Model B
	B (SE)	B (SE)
Intercept	4.10 (0.07)^∗∗∗^	4.08 (0.07)^∗∗∗^
Fluency		0.27 (0.07)^∗∗∗^
AUT task (-0.5: non-switch, +0.5: switch)	-0.22 (0.14)	-0.01 (0.14)
Habitual language switching	0.10 (0.07)	0.13 (0.07)
AUT task ^∗^ habitual language switching	0.37 (0.14)^∗∗^	0.49 (0.14)^∗∗∗^

^∗∗^*p < 0.01; ^∗∗∗^*p* < 0.001*.

**FIGURE 2:**
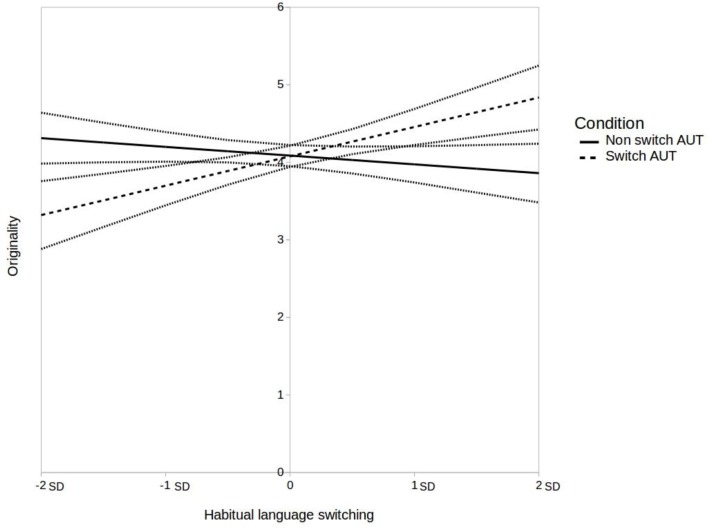
Interaction of language switching condition and habitual language switching on originality scores, controlling for fluency scores (Model B). 95% confidence intervals are represented by gray lines.

This analysis yielded a significant AUT condition x habitual language switching interaction effect, *B* = 0.49, *SE* = 0.14, *t* = 3.58, *p* < 0.001. The interaction effect is shown in **Figure 2**. Simple slope analyses were conducted to further investigate the interaction effect. As expected, bilinguals who switch languages more often (i.e., at +2 SD from the mean) tended to have higher originality scores in the switch condition, than their counterparts in the non-switch condition, *B* = 0.97, *SE* = 0.32, *t* = 3.09, *p* = 0.003. Bilinguals who switch languages less often (i.e., at -2 SD from the mean) tended to have lower originality scores in the switch condition, than their counterparts in the non-switch condition, *B* = -0.99, *SE* = 0.30, *t* = -3.28, *p* = 0.001.

We also proceeded to additional exploratory simple slope analyses in order to provide a more complete description of the interaction effect. For these unplanned comparisons, we applied a Bonferroni correction and set the significance threshold at 0.01. The analyses revealed that in the non-switch condition, there was no association between habitual switching and originality scores, *B* = -0.11, *SE* = 0.09, *t* = -1.32, *p* = 0.191. Only in the switch condition, there was a positive relationship between habitual language switching and originality scores, *B* = 0.38, *SE* = 0.11, *t* = 3.60, *p* < 0.001. This suggests that habitual switching is only beneficial to originality in a context of active language switching.

## Discussion

Our aim was to investigate the effect of language switching on creative production in an AUT (Guilford, 1967) among bilinguals. Our results indicated that the more bilingual individuals are used to switch languages, the higher their originality scores in the switch AUT compared to in the non-switch AUT. Conversely, the less bilinguals are used to switch languages, the higher their originality scores in the non-switch AUT compared to in the switch AUT. These findings are in line with our theoretical expectations. Language switching seems to be better than not switching for the originality scores of individuals who are used to switching languages in their daily life. For bilinguals who are not used to engage in language switching, the reverse seems to be the case, with not switching languages during the AUT’s leading to ideas that are more original than switching during the AUT’s.

Our study extends previous research on language switching and creativity by using an experimental design to investigate the effect of within task language switching on the originality of ideas. Contrary to Kharkhurin and Wei (2015), we did not find that bilinguals who switch languages more often in daily life find ideas that are more original than their counterparts in traditional (i.e., non-switch) AUT’s. We only found an effect of habitual language switching in the switch condition. In Kharkhurin and Wei’s (2015) study, bilinguals filled in questions about their habits in terms of language switching prior to performing the divergent thinking tasks. In our study, the order of tasks was reversed. It is therefore possible that Kharkhurin and Wei’s (2015) design activated a language switching mindset while performing the tasks, by making participants aware of their language switching habits. Replicating Kharkhurin and Wei’s (2015) study with a counter-balanced design could shed light on the reasons of the observed differences with our own findings.

An unexpected finding in our study is that fluency scores were not lower for all participants in the switch AUT compared to the non-switch AUT. Only at higher levels of daily language switching this effect was found. This seems to refute our idea that language switching overall negatively impacts fluency. It means that at higher levels of language switching in daily life, participants in the switch AUT produced fewer ideas (than participants in the non-switch AUT), yet their ideas were more original on average. This is an interesting finding that deserves further study. More specifically, one could wonder whether participants filtered out non-original ideas, whether the originality level of each individual idea went up, or whether both processes were combined. Additionally, our study does not provide evidence regarding the direction of the effect. More specifically, we cannot distinguish whether creativity was particularly stimulated, or whether it was inhibited by the (non) switch manipulation.

To thoroughly answer these questions, further research is needed with a design that allows the generation of more ideas – using AUT’s in which participants have more time to generate ideas (e.g., 10 min instead of 2 min in our study) – and which includes a baseline condition. With a larger number of ideas, it would be possible to examine the distribution of the originality of ideas in the switching and non-switching AUT, and test how the distribution of the originality of ideas among participants who are used to switch differs from the distribution of the originality of ideas among participants who are not used to switch. With a baseline condition, one could determine whether language switching (vs. not switching) stimulates and/or inhibits creativity. Nevertheless, our findings are valuable from a practical point of view, by underlining the importance of respecting individuals’ habitual use of their two languages. It seems that to get the most of an individual’s creativity in the short run it is important to put the individual in a task whose characteristics match the individual’s habitual state regarding language switching.

Our study has other limitations. First, we did not measure the language proficiency of participants. It is possible that differences in language proficiency between bilinguals who switch languages more often or bilinguals who switch languages less often could partly explain the results we found. A second limitation is related to the fact that we did not control for intelligence of participants. Beaty and Silvia (2012) showed indeed that highly intelligent individuals do not need to go through many unoriginal ideas to arrive at more original ideas. It is therefore possible that our results concerning fluency and originality are partly due to the fact that, by chance, participants who often switch languages in the switch condition had a higher level of intelligence than other participants. This is the reason why replications of our study that control for the intelligence level of participants are needed. A third limitation is related to the fact that bilinguals who switch languages more often might also be more often exposed to several cultures, in addition to purely engaging in language switching. Being exposed to multiple cultures also has an impact on creativity (Storme et al., 2015; Çelik et al., 2016; Cheung et al., 2016). Exposure to multiple cultures could therefore partly explain our results. Replicating our study while controlling for exposure to multiple cultures could help better delineate the relative contribution of habitual language switching and exposure to multiculturalism to the effects that we found. A final limitation is the fact that we used a between-subjects design. Although the allocation of participants in the condition was random, it is possible that participants in the switch and the non-switch AUT conditions systematically differed on some aspects that could explain the differences that we found between the two conditions. Using a within-subjects design would limit the possible interferences of individual differences that we did not control in the analyses.

Debates on bilingualism and language switching on young bilinguals’ development and school performance may well increase given the current societal developments of increasing cultural and linguistic diversity at schools. Research on this subject is thus very important. Our findings will hopefully spur future research into investigating more in depth the effects of language switching on cognitive processes like creativity, which have great individual and societal value.

## Ethics Statement

This study was carried out in accordance with the recommendations of Paris Descartes University with written informed consent from all subjects.

## Author Contributions

MS, PC, AC, and BF designed the study, collected and analyzed data, and wrote the article. HH and TL supervised the project.

## Conflict of Interest Statement

The authors declare that the research was conducted in the absence of any commercial or financial relationships that could be construed as a potential conflict of interest.

## Footnotes

1 The main analysis on originality scores without fluency as a control did not change the pattern of results, but did weaken the effect (see Model A in **Table 1**), indicating a suppression (Hayes, 2009).
